# APDB: a database on air pollutant characterization and similarity prediction

**DOI:** 10.1093/database/baad046

**Published:** 2023-07-14

**Authors:** Eva Viesi, Davide Stefano Sardina, Ugo Perricone, Rosalba Giugno

**Affiliations:** Department of Computer Science, University of Verona, Strada le Grazie 15, Verona 37134, Italy; Molecular Informatics Unit, Ri.MED Foundation, Via Filippo Marini 14, Palermo 90128, Italy; Molecular Informatics Unit, Ri.MED Foundation, Via Filippo Marini 14, Palermo 90128, Italy; National Biodiversity Future Center (NBFC), Piazza Marina 61, Palermo 90133, Italy; Department of Computer Science, University of Verona, Strada le Grazie 15, Verona 37134, Italy; National Biodiversity Future Center (NBFC), Piazza Marina 61, Palermo 90133, Italy

## Abstract

The World Health Organization estimates that 9 out of 10 people worldwide breathe air containing high levels of pollutants. Long-term and chronic exposure to high concentrations of air pollutants is associated with deleterious effects on vital organs, including increased inflammation in the lungs, oxidative stress in the heart and disruption of the blood–brain barrier. For this reason, in an effort to find an association between exposure to pollutants and the toxicological effects observable on human health, an online resource collecting and characterizing in detail pollutant molecules could be helpful to investigate their properties and mechanisms of action. We developed a database, APDB, collecting air-pollutant-related data from different online resources, in particular, molecules from the US Environmental Protection Agency, their associated targets and bioassays found in the PubChem chemical repository and their computed molecular descriptors and quantum mechanics properties. A web interface allows (i) to browse data by category, (ii) to navigate the database by querying molecules and targets and (iii) to visualize and download molecule and target structures as well as computed descriptors and similarities. The desired data can be freely exported in textual/tabular format and the whole database in SQL format.

**Database URL** http://apdb.di.univr.it

## Introduction

Air pollution is a mixture of hazardous substances, including gases, organic compounds and metals, from both human-made and natural sources [https://www.niehs.nih.gov/health/topics/agents/air-pollution/ (7 March 2023, date last accessed)]. Chronic exposure to air pollution appears to cause deleterious effects on human health, affecting lung and heart functions, as well as generating alterations in brain cognitive functions that may potentially increase risk factors for neurodegenerative disorders such as dementia, Alzheimer’s and Parkinson’s diseases (1, 2).

Thus, in the endeavour to deeply understand the molecular and cellular mechanisms responsible for the connection between vital organs’ health and air pollution, it is necessary to accurately collect, organize and chemically characterize pollutant molecules.

A comprehensive list of air pollutants is provided by the Environmental Protection Agency (EPA), in particular, the EPA has released the SPECIATE database, a curated repository of speciation profiles of air pollution sources that describes the chemical composition of organic gas, particulate matter (PM) and other pollutants emitted by these sources [https://www.epa.gov/air-emissions-modeling/speciate-0 (7 March 2023, date last accessed)]. Supplied chemical species include six air pollutants identified as ‘criteria’, namely ground-level ozone, PM, carbon monoxide, lead, sulphur dioxide and nitrogen dioxide. The EPA, in regulating emissions of hazardous air pollutants, provided an original list later modified through rule-making to include 188 hazardous air pollutants, which are classified as ‘HAPS’ in SPECIATE [https://www.epa.gov/haps/initial-list-hazardous-air-pollutants-modifications (7 March 2023, date last accessed)].

Other sources of air pollution information are the Agency for Toxic Substances and Disease Registry Portal, collecting details on adverse health effects due to exposure to hazardous substances [https://wwwn.cdc.gov/TSP/index.aspx (7 March 2023, date last accessed)], the Risk Assessment Information System [https://rais.ornl.gov/tools/tox_profiles.html (7 March 2023, date last accessed)], containing toxicity profiles of different chemical species, and the Hydrosil International Ltd website [https://hydrosilintl.com/resources/pollutants (7 March 2023, date last accessed)], a leading producer of odour, water, hazardous air pollutants and volatile organic compounds’ control filtration products.

Molecular structures of air pollutants can be represented in the most recognized chemical-data file formats, e.g. the SDF format (Structure Data Format), a formatted ASCII file widely used to store and exchange comprehensive information about the chemical structure of molecules, including their atom types, bond types, 3D coordinates and other relevant properties, and the SMILES format (Simplified Molecular-Input Line-Entry System), a compact ASCII string that encodes the connectivity of atoms in a molecule in a standardized manner which makes it particularly suitable for database searching [http://www.structuralchemistry.org/pcsb/capp_cdf.php (7 March 2023, date last accessed)].

Molecules of interest can be further annotated using several online chemical sources, such as PubChem (3), a publicly available resource of chemical information maintained by the National Center for Biotechnology Information, which includes the structure, properties and biological activities of 120 million chemical compounds, including small molecules, peptides and larger molecules like proteins and nucleic acids. It also annotates compounds with external resources and allows you to browse the chemical information through a user-friendly interface making it a valuable resource for researchers. ChEMBL (4) and ChEBI (5) are both supported by the European Molecular Biology Laboratory’s European Bioinformatics Institute. The former is an open, manually curated and high-quality chemical database of 2 million bioactive molecules and their targets, hence primarily useful for drug discovery and pharmacology studies. The latter covers a wider range of chemical entities, including small molecules, peptides and other compounds. It contains a freely accessible ontology that provides their classification, nomenclature and chemical properties.

The above web resources provide different molecular identifiers, among them the Chemical Abstracts Service (CAS) Registry Number that uniquely identifies substances, the InChIKey (International Chemical Identifier), a textual identifier encoding molecular information, the canonical SMILES, a unique structural in-line representation for a molecule and the molecular formula (e.g. C_2_H_3_Cl_2_F). Moreover, these databases contain the collections of bioactivity and toxicity data associated with small molecules, which can be easily accessed through public Application Programming Interfaces and processing utilities in batch mode.

Molecules can be characterized through a wide range of structural and physicochemical properties, also called molecular descriptors. Descriptors can be calculated starting from two-dimensional (2D) structures [e.g. 2D autocorrelation indices by Moreau–Broto (ATS), Moran (MATS) and Geary (GATS) algorithms or topological charge indices (6)] and from three-dimensional (3D) structures (e.g. charged partial surface area (7) or Weighted Holistic Invariant Molecular indices (8)). Among properties based on quantum mechanics (QM), the orbital energies of the highest occupied molecular orbital (HOMO) and the lowest unoccupied molecular orbital (LUMO) [https://chem.libretexts.org/Bookshelves/General_Chemistry/General_Chemistry_Supplement_(Eames)/Molecular_Orbital_Theory/Frontier_MOs%3A_An_Acid-Base_Theory (7 March 2023, date last accessed)], which describe the tendency of a molecule to behave as a nucleophile or an electrophile, are the most known. QM methods accurately describe the behaviour of the electrons in atoms and molecules based on the Schrödinger equation that governs the wave function representing all the properties, e.g. momentum, time, position and spin, of a particle in a quantum system [http://hyperphysics.phy-astr.gsu.edu/hbase/quantum/schr.html (7 March 2023, date last accessed)].

All the aforementioned molecular properties and descriptors can be computed from open-source chemoinformatics libraries, RDKit (9), PaDEL-Descriptor (PaDEL for the sake of brevity) (10) or the Chemistry Development Kit (11), as well as proprietary licence software, such as Jaguar from Schrödinger company (12). In particular, Jaguar software, an *ab initio* quantum chemistry programme that applies to both organic and inorganic chemistry, is used to calculate molecular properties (e.g. molecular orbitals and electron density) and QM properties (e.g. vibrational frequencies, multipole moments, polarizabilities, enthalpies, and entropies). PaDEL instead is used to compute physicochemical properties, a wide range of 2D and 3D molecular descriptors and fingerprints. The fingerprint of small molecules is a binary or a count string representation that can be fragment-based, encoding the presence or absence of specific structural features or patterns, e.g. MACCS (Molecular ACCess System) keys (13) or the E-state fingerprint by Hall and Kier (14), or hash-based, encoding bond paths up to a fixed length [e.g. Chemistry Development Kit (CDK) fingerprint].

Molecular descriptors and fingerprints are widely used for chemical substructure search, molecular similarity calculation, clustering of molecules and so forth [https://www.daylight.com/dayhtml/doc/theory/theory.finger.html (7 March 2023, date last accessed)]. Depending on the type of descriptor, several measures can be used to derive similar molecules. For example, for numeric vectors (e.g. 2D autocorrelation indices or E-state indices), the similarity is the complement of the normalized Euclidean distance or just the cosine similarity between them (15). Usually, for binary descriptors (e.g. 2D fingerprints and atom pairs), the mostly used similarity metrics are the Tanimoto coefficient or the Tversky index (16), defined as no. of bits = 1 in both sets/no. of bits = 1 in either set. Molecular similarity has been demonstrated to be relevant in drug discovery campaigns to find new molecules or to repurpose drugs (17) and can be likewise applied in toxicology to infer chemical toxicity based on the similarity of a molecule with known toxicants. This is useful also in comparing pollutants in order to let scientists deeply analyse and understand the putative toxic effect of air pollution.

**Figure 1.**
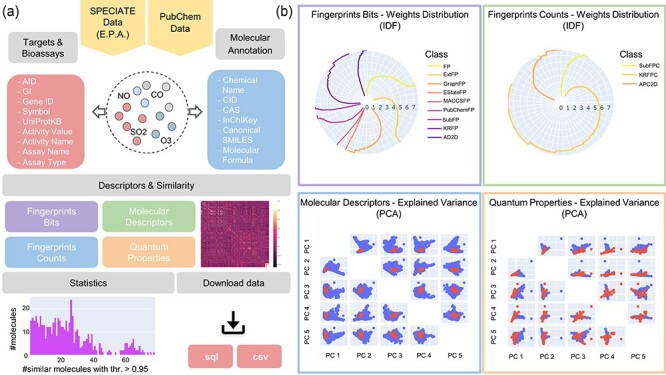
An APDB overview. (**a**) The online resources, the collected molecular annotations, targets and bioassays, the computed molecular descriptors and similarities, the data statistics and the download functionalities. (**b)** The IDF weights distribution of fingerprints bits and counts and the first five principal components of molecular descriptors and quantum properties.

Here, we built a database, APDB, which takes strength from all the information available in the aforementioned resources to give a comprehensive and easy-to-use platform for the analysis of pollutant molecules (Figure 1a). Publicly available chemical databases report large-scale molecules to be used for different purposes, e.g. drug discovery, quantitative structure-activity relationship and quantitative structure-property relationship. Usually, molecular descriptors are also provided (18), or they can be computed for the investigated chemical structures (19). However, these results do not take into account energy minimization or consider only minimum local energy. Unlike other resources (19–22), we focused on similarities between air pollutants by using molecular and quantum mechanical descriptors which analyse physical properties at the atomic or subatomic scale. Moreover, APDB specifically provides chemical space visualization and similarity search of air pollutants by leveraging graph embeddings to create air pollutant signatures.

**Figure 2.**
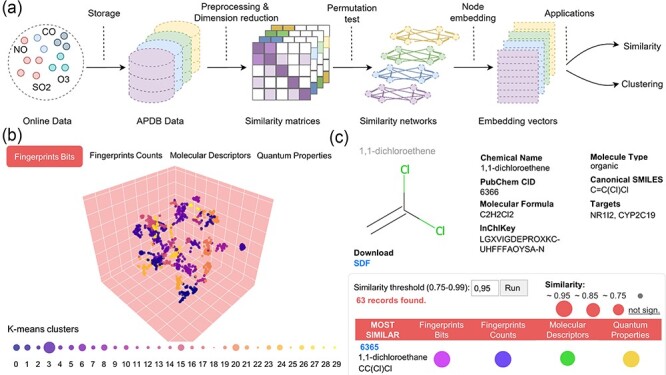
Data preprocessing and similarity analysis. (**a**) From data collection and storage to data preprocessing and similarity calculation. (**b**) *k*-means clustering of similarity spaces. (**c**) The panel of similar molecules to a query molecule (default threshold is 0.95; if no similar molecules are found, those with the closest threshold are returned).

APDB is enriched with collected molecular annotations, the computed 2D, 3D and QM descriptors (as shown in Figure 1b), the identified targets and bioassays and the predicted similar molecules. Molecular descriptors and properties are exploited to derive molecular similarities (Figure 2). Indeed, each identified chemical dataset is turned into a numeric matrix of molecular features by latent semantic indexing (LSI) or principal component analysis (PCA), and similar molecules are retrieved by applying the most common similarity and dissimilarity measures. The significance of the computed similarities is assessed by a permutation test. As a last step, a node embedding algorithm is applied to the obtained similarity networks to explore the most similar molecules given a threshold. All data are freely accessible via a web application that allows database searches and data visualization and download.

The rest of the paper is organized as follows. The section ‘Materials and methods’ details the proposed methodology providing information on (i) collected molecular data in APDB, (ii) computed quantum properties, (iii) calculated molecular descriptors and fingerprints, (iv) derived molecular similarity, (v) implementation of the web interface and (vi) overview of the database. The section ‘Utility and discussion’ describes case studies and discusses obtained results. The section ‘Conclusion’ summarizes key points and gives suggestions on possible applications.

## Materials and methods

### The molecular data in APDB

The APDB database contains a collection of air pollutant molecules from the US EPA website, which provides the list of SPECIATE data [https://www.epa.gov/air-emissions-modeling/speciate-0 (7 March 2023, date last accessed)] that also incorporates the list of hazardous air pollutants [https://www.epa.gov/haps/initial-list-hazardous-air-pollutants-modifications (accessed 7 March 2023, date last accessed)] classified as ‘HAPS’. SPECIATE 5.1 (June 2020) is released both in Microsoft Access® and in the SPECIATE Data Browser.

We used this resource as a starting point for building content in APDB; specifically, we exported the ‘SPECIES_PROPERTIES’ table containing molecule information such as CAS number, species name, SMILES notation and molecular formula. We kept only one chemical name per molecule, eliminating synonyms and removing duplicated species, CAS numbers and missing or corrupted SMILES strings.

In APBD, molecules are annotated with PubChem’s molecular identifiers and properties, such as PubChem Compound Identification (CID), CAS, InChIKey, canonical SMILES and molecular formula, retrieved via PubChem’s PUG REST service querying by SMILES string (23). Each queried SMILES was associated with a single compound in PubChem, resulting in a total of 1830 molecules from an initial set of about 2800 species.

To determine the molecule type, i.e. organic or inorganic, we used a web-based application named ClassyFire (24), which hierarchically classifies chemical entities. We employed RDKit (9), an open-source chemoinformatics toolkit, to list all the unique atoms present in every molecule and generate the 2D molecular structures starting from the canonical SMILES. For each molecule, its associated targets found in the PubChem BioAssay database (25) are reported. We accessed bioassay data through PubChem’s RESTful interface querying by PubChem CID and searching for the word ‘active’ in the assay description. We annotated each target with the gene symbol and UniProtKB identifier using the AnnotationDbi R/Bioconductor package (26), keeping only human genes. Overall, the number of molecules with associated targets is 537.

### The calculation of QM properties

QM properties were computed using Jaguar (12) computational program on Maestro, a graphical user interface that provides access to Schrödinger’s software and allows displaying and manipulating chemical structures [https://www.schrodinger.com/products/maestro (7 March 2023, date last accessed)], while additional molecular properties, descriptors and fingerprints were calculated with the PaDEL-Descriptor software (10).

The computation of quantum mechanical descriptors comprises two main tasks: (I) geometry optimization and (II) single-point energy calculation. Canonical SMILES were imported in Maestro in order to clean them up and check the correctness of atom connectivity, bond orders and desalting, using the LigPrep module [https://www.schrodinger.com/products/ligprep (7 March 2023, date last accessed)], which generates an energy-minimized and accurate 3D representation of a molecule, i.e. its most stable conformation, allowing to avoid issues with the molecular structures and geometries. We applied the OPLS4 molecular mechanics force field (27) and selected the Epik tool for pKa prediction to produce possible ionization and tautomeric states at pH 7.0 ± 2.0. pKa is a property that describes how acidic or basic a chemical entity is by reflecting the ionization state, i.e. the charge, and the tautomeric states, i.e. the interconvertible structures, of a molecule in solution at a given pH value (28). LigPrep was not applied to molecules containing metallic elements, small compounds and ions. The output SDF file [http://www.structuralchemistry.org/pcsb/capp_cdf.php (7 March 2023, date last accessed)] reports information about the ionization state, formal charge and added hydrogen. To improve the readability, the details of the output fields are given in Supplementary Table S1.

Only one structure for each molecule was selected for further optimization (as described later) according to the lowest conformational energy, state penalty, ionization penalty and highest tautomer probability.

The preprocessed molecules were subsequently given in input to Jaguar. We first performed a default-level optimization task to converge the molecular structure to a minimum geometry and then we ran a single-point energy calculation for computing the quantum properties. Both steps require the choice of density functionals and basis sets. The density functional theory (DFT) is a widely employed computational method to calculate the electronic, structural and magnetic properties of atoms and molecules. This theory aims to quantitatively understand the properties of a QM system by solving the Schrödinger equation based on the electron density, which represents the probability of finding an electron at a specific region around atoms and molecules (29). Several density functionals are available in Jaguar, so we decided to apply the traditional hybrid density functional B3LYP (30) to all molecules to perform both tasks. Like many quantum chemistry software programs, Jaguar employs basis sets (sets of basis functions) consisting of Gaussian-type atomic orbitals to represent the electronic wavefunction or electron densities used in the DFT. The standard basis set in Jaguar is called 6-31G and covers the elements from hydrogen to argon (12); therefore, we used the 6-31G** basis set to perform geometry optimization of all molecules containing H–Ar and non-metal elements (except for iodine).

For ‘SCF’ convergence, which is a self-consistent field method to investigate electronic structure configurations within the DFT, we selected the ‘Quick’ accuracy level for geometry optimization and the ‘Accurate’ for single-point calculation, while we left the initial guess as ‘Atomic overlap’. We kept the default convergence criteria and methods, except in some particular cases in which we increased the number of maximum iterations to help convergence. In the ‘Solvation’ settings, we selected the ‘None’ option as the solvent model since we did not analyse molecules in solution, but in gas phase.

After coordinate optimization, it is common to use larger basis sets, i.e. having more functions per atom, in a single-point calculation to compute QM properties. In the work by Bochevarov *et al.* (12), the authors recommend using cc-pVTZ(-f) for accurate computations of the energy; thus, we applied this basis set to all molecules containing elements from hydrogen to argon and remaining non-metals, after geometry optimization. For the molecules not converging with cc-pVTZ(-f), namely long-chain fatty acids, we used again 6-31 G** in the single-point task and increased the number of maximum iterations to 1000. For metal atoms beyond argon, it is strongly recommended the usage of the LACVP** basis set for geometry optimization and LACV3P** subsequent energy evaluation; therefore, we applied these basis sets to the different metals and molecules containing metallic elements beyond argon. For a few compounds not converging in optimization, we directly performed single-point energy calculation changing the atom-level settings for every single atom, i.e. we chose cc-PVTZ(-f) or LACV3P** depending on the atom type [e.g. for hydroxyapatite (HAp), potassium carbonate and chrysotile]. As described in the reference manual (31), since lanthanides are covered by the CSDZ basis set, we applied CSDZ** to directly execute the single-point energy task. The ERMLER2 ECP basis set instead is supported by both lanthanides and actinides; therefore, we used ERMLER2** for actinides elements and for those lanthanides not converging with the CSDZ basis set. We still selected ‘Accurate’ as the accuracy level and increased the number of maximum iterations when needed.

Molecular properties selected for geometry optimization and single-point calculations are provided in Supplementary Table S2. Quantum-chemical properties written to the output structure file (*.sdf*) are listed and detailed in Supplementary Table S3.

### The molecular descriptors and fingerprints

The output structure files from single-point energy calculations were used to compute physicochemical properties, 2D and 3D descriptors and fingerprints with PaDEL. It currently computes 797 types of descriptors (663 1D, 2D descriptors and 134 3D descriptors) and 12 classes of fingerprints (10) (note that we did not compute 3D descriptors for metal-containing inorganic molecules due to the lack of reliable 3D information). The obtained 1D descriptors mainly represent information computed from the molecular formula, while the more complex 2D descriptors describe molecular features regarding the size, morphology and electron distribution in the molecule; finally, 3D descriptors represent properties related to the 3D molecular conformation (32). Common 1D descriptors are atom and bond counts and types, and molecular weight, while remarkable 2D descriptors are the Ghose–Crippen octanol-water coefficient (*A*Log*P*) expressing lipophilicity, the Ghose–Crippen molar refractivity (AMR) that measures dispersive interactions (33), the Moreau–Broto (ATS), Moran (MATS) and Geary (GATS) autocorrelation indices, i.e. topological descriptors encoding both molecular structure and physicochemical properties (such as mass, van der Waals forces and electronegativities) of a molecule (34) and the atom-type electrotopological state (E-state) indices, which combine the electronic state of an atom with its topological context within a molecule (14). Examples of 3D descriptors obtained from the 3D structure of molecules are the polar and non-polar surface area (35); moreover, autocorrelation can also be calculated from 3D molecular geometry.

Molecular fingerprints are a way of representing a molecule as a vector whose components encode the presence/absence, or the counts, of a specific functional group, scaffold or feature in the molecular graph (36). We obtained different classes of fingerprints from PaDEL, namely the CDK and CDK-extended fingerprints (FP and ExtFP) (11), examples of path-based hashed fingerprints that encode paths of length 8 into a 1024-bit array; the E-state fingerprint, a 79-bit array that represents the presence/absence of the 79 E-state atom types defined by Kier and Hall (14); the CDK graph only fingerprint, which is a specialized version of the CDK FP; the MACCS fingerprint that indicates the presence/absence of 166 structural features called MACCS keys (13); the PubChem fingerprint, a 881-bit array encoding molecular fragment information [https://ftp.ncbi.nlm.nih.gov/pubchem/specifications/pubchem_fingerprints.pdf (7 March 2023, date last accessed)]; the substructure fingerprint that represents the presence or count of 307 SMARTS patterns describing atomic and bond properties [https://www.daylight.com/dayhtml/doc/theory/theory.smarts.html (7 March 2023, date last accessed)]; the Klekota–Roth fingerprint, a 4860-bit array that describes the presence or count of chemical substructures defined by Klekota and Roth (37) and the 2D atom pairs fingerprint encoding the presence or count of atom pairs at various topological distances in the molecular bond graph (e.g. F–F pair at distance 4).

### The molecular similarity

All the described molecular properties and descriptors were used to calculate molecular similarities in the APDB as outlined in Figure 2a.

Following the direction of Duran-Frigola *et al.* (20), APDB data were divided into four identified ‘chemical spaces’: (i) fingerprints bits, (ii) fingerprints counts, (iii) molecular descriptors and (iv) quantum properties. In addition, single elements were filtered from the last two spaces to be analysed separately. Each dataset was turned into a numeric matrix having molecules as rows and features as columns. In particular, term frequency–inverse document frequency (TF–IDF) transformation was applied to discrete numeric data represented by bits and counts associated with each molecule, and dimensionality reduction, called LSI based on singular-value decomposition, was performed on the resulting TF–IDF matrix by keeping the number of components explaining 80% of the variance (38). For continuous numeric data, PCA (39) for dimensionality reduction was applied to the preprocessed matrices after managing missing values and scaling data, by keeping 80% or 90% of the variance depending on the number of input features. Similar molecules were derived by primarily applying cosine similarity to the reduced numeric matrices. To assess the significance of the computed similarities, a permutation-based test was performed on the low-dimensional matrices by shuffling values within rows and by comparing the expected pairwise similarity matrix with the true similarity matrix for a given number of permutations (default 1000). Subsequently, similarity networks were built from statistically significant pairwise similarities, i.e. with a *P*-value of <0.05 or 0.01. The final features associated with each molecule are low-dimensional vectors obtained by running the ‘Node2Vec’ (40) node embedding algorithm on each similarity network. In particular, we used 36 dimensions for molecular fingerprints and descriptors, and 9 dimensions for quantum properties. The obtained embedding model, which maximizes the likelihood of preserving the original network structure, was used to explore the most similar molecules, given a similarity threshold. Embedding vectors were also used to cluster molecules into communities or groups of similar compounds by *k*-means (41) (Figure 2b) or agglomerative clustering (42).

Finally, we performed a similarity assessment by computing the association between the number of common targets found between similar molecules and between not similar molecules. Similar molecules are those with a high similarity in the embedding space (e.g. >0.96), while not similar molecules are those with a low similarity (e.g. <0.46). Through a chi-squared test, we found that the association between being similar or not similar and having common targets was statistically significant. Moreover, we observed that the group of similar molecules had slightly higher odds of having common targets than not similar molecules.

### Web interface implementation

APDB is a relational database implemented in PostgreSQL with a web application developed using Python (version 3.10.4) and Flask (version 2.1.2) (43). The front end was designed using the HTML and CSS languages and the Bootstrap framework. The application was developed with Docker on Ubuntu 18.04 server and consists of two containers, one with the main Flask application and one with the PostgreSQL database.

RDKit (9) was used to dynamically generate the 2D molecules’ structures, and 3Dmol.js (44) was embedded to visualize high-resolution target 3D structures from the Protein Data Bank (PDB) (45).

### Web interface overview

The main APDB interface modules are Home, Statistics and Downloads/Contacts. These allow (i) to navigate the database by searching for molecules, targets and bioassays, descriptors and similarities; (ii) to visualize data statistics and (iii) to download data.

#### Home

Through the Home section, the user is directed to subsequent pages where one can browse molecules, targets and bioassays, descriptors and similarities.

- *Molecular annotations*: Molecules are specified by using the chemical name, CID, CAS, InChIKey, canonical SMILES and molecular formula from PubChem. Molecule structures can be filtered by clicking on one periodic table element. By pressing the download button, users can obtain a CSV file with the whole table or with the selected items from the column checkboxes, the periodic table or the search form. Molecules can be searched by all their listed identifiers. By clicking on a CID entry, users are redirected to the panel of similar molecules.
- *Targets and bioassays*: Bioassays are specified through the assay ID, associated CID, activity value in µm, activity name, assay name, assay type and PubMed ID. Targets are inserted by their GenInfo Identifier, gene ID, symbol and UniProtKB. By pressing the download button, users can obtain a CSV file with the whole table or with the selected items from the column checkboxes or the search form. Targets can be searched by all their listed identifiers. By clicking on a UniProtKB entry, users are redirected to the target panel containing information on the searched target. If present, the optimized PDB file generated with 3Dmol.js can be visualized and downloaded through the ‘PDB’ button; otherwise, users can download and explore the predicted protein structure on the AlphaFold page. Molecules associated with the searched target can be downloaded as a CSV file by pressing on the download icon. By clicking on a CID entry, users are redirected to the panel of similar molecules.
- *Molecular descriptors*: Descriptors are illustrated through an interactive radar chart representing the distribution of the IDF weights of all classes of fingerprints bits and counts and a scatter plot matrix with the first five principal components of molecular descriptors and quantum properties. By pressing on the download icon, users can obtain a CSV file with the corresponding descriptor table.
- *Molecular similarities*: Similarity spaces are illustrated through an interactive scatter plot of *k*-means clusters projected to 3D with t-distributed Stochastic Neighbor Embedding (t-SNE) (Figure 2b). The legend below shows the number of similar molecules found for each cluster. By pressing on the download icon, users can obtain a .zip file with the corresponding embedding model from ‘Node2Vec’ (note that molecular descriptors and quantum properties also include the model for single elements). Similar molecules can be retrieved by inserting the CID or InChIKey of a molecule of interest in the search area. The molecule similarity panel (Figure 2c) shows molecular info together with the 2D structure generated with RDKit and the list of similar molecules ranked by the number of intersecting spaces and average similarity. Users can change the similarity threshold in the range of 0.75–0.99 (default 0.95); if no similar molecules are found, the application automatically decreases the threshold until at least one similar molecule is returned. The ‘SDF’ button allows downloading the optimized SDF file for the searched molecule. Similar molecules can be downloaded as a CSV file by pressing on the download icon in the table header.

#### Statistics

The Statistics section illustrates some interactive charts on data in APDB. A pie plot shows the percentage of organic and inorganic molecules. A histogram plot represents the count of molecules with a specific number of targets. A set of histograms shows the distribution of similar molecules at different similarity thresholds. A table describes the number of entries in APDB.

#### Documentation

Through the ‘Documentation’ section, the user can have an overview of the data contained in the APDB and a quick guide to the main sections and utilities.

#### Downloads/Contacts

In the ‘Downloads/Contacts’ section, the whole database can be dumped as a compressed archive .zip or in .sql format (except for molecular descriptors and fingerprints which can be downloaded in .csv format from the dedicated page). Search results (i.e. by molecule, target and similarity) can be downloaded in .csv format through the corresponding ‘Download’ buttons.

## Results and discussion

The user can easily access and browse APDB molecular information through the appropriate sections and search for a query molecule to get similar compounds, known biological targets and related chemical features. For each chemical entity, the results represent the specific contribution of each chemical space in computing similar molecules as described in the section ‘The molecular similarity’.

### Case studies

**Figure 3.**
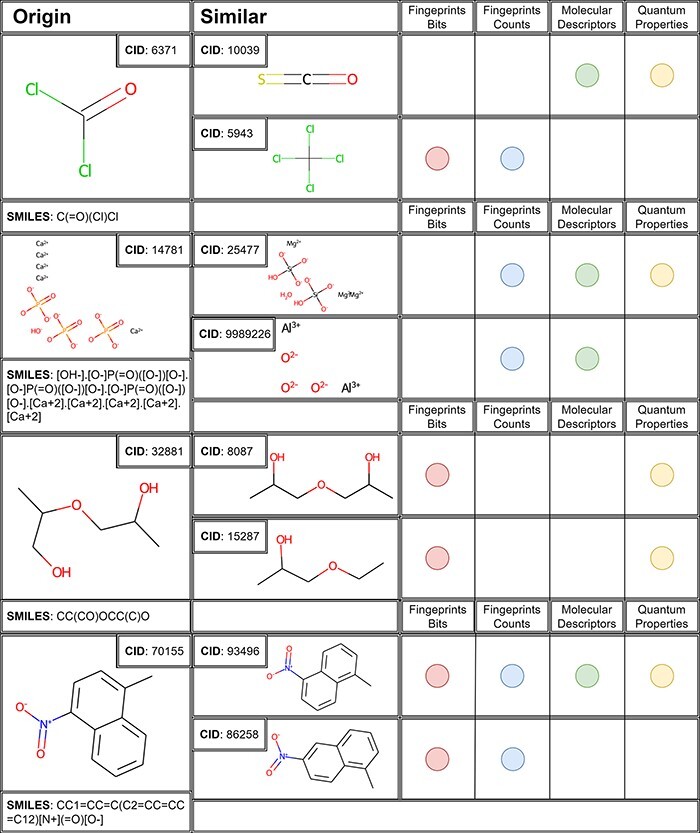
The first two similar molecules for each case study molecule with a similarity threshold of >0.96 in the corresponding space.

We selected the most representative molecules to illustrate the features and usability of APDB, e.g. 2-(2-hydroxypropoxy)propan-1-ol, 1-methyl-4-nitronaphthalene for organic and HAp, and phosgene for inorganic compounds (Figure 3). These four molecules were selected according to chemical features and complexity. The two organic compounds are examples of aromatic, cyclic, linear substructures and several functional groups. Inorganic compounds were chosen because they represent the low and high complexity of typical inorganic molecules. 2-(2-Hydroxypropoxy)propan-1-ol is not a common compound in industry or laboratory; however, it could potentially be used in the synthesis of other compounds or as a solvent.

1-Methyl-4-nitronaphthalene is a nitro derivative of naphthalene, classified as a nitroaromatic compound which in turn has been used as an intermediate in the synthesis of other organic compounds, such as dyes, pesticides and pharmaceuticals (46). Indeed, there is concern about its potential toxicity and carcinogenicity (47).

Both compounds are not widely studied and their analysis can potentially shed light on negative effects on human life.

HAp is a naturally occurring mineral form of calcium phosphate. It is the main mineral component of bones and teeth in living organisms and is insoluble in water and organic solvents. HAp has a number of unique properties that make it useful in a variety of industrial and medical applications. Due to its chemical similarity to the mineral component of bone, it has been used as a bone substitute material in orthopaedic and dental applications. It is also used as a coating material on metal implants to promote bone growth and improve the bonding between the implant and the surrounding bone.

Phosgene is a poisonous and colourless gas primarily used as a constituent in many pharmaceutical and organic industries. Once inhaled, it produces a dose-dependent toxic effect by acylating several enzymes related to energy metabolism, which in turn causes a breakdown of the blood–air barrier resulting in the clinical manifestation of pulmonary oedema (48).

Analysing the results of case studies queries, it is noteworthy how every chemical space of similarity shows interesting and complementary similarity aspects between molecules. Thus, the consultation and comparison between different chemical spaces offer the user the possibility to analyse similarities from different points of view. Fingerprints spaces will give back molecules containing similar fragments within the chemical structure, whereas molecular descriptors and quantum mechanical descriptors will propose molecules with structural and reactivity similarities.

For example, analysis of phosgene results (Figure 3) shows carbonyl sulphide (CID 10 039) as the most similar compound, due to the presence of the carbonyl moiety detected by molecular and QM properties as the most similar in terms of reactivity and chemical composition. Interestingly, these two gases have been reported to share toxicity mechanisms and they have been recently also evaluated as comparable for the HOMO–LUMO reactivity towards aromatic compounds (49). Fingerprints, instead, find other similar fragments such as the chlorine atoms connected to a central carbon atom, contained in the carbon tetrachloride (CID 59 430). Also in this case, the similarity tool offered important data, considering that the hepatotoxic effect of carbon tetrachloride is mediated by its transformation in tissues, where its peroxidation generates phosgene (50). This case study demonstrates how the similarity tool can be useful for direct molecular similarity or to suggest toxicity mechanisms behind a molecular structure otherwise neglected without considering possible metabolites.

For the other inorganic compound chosen as a case study, HAp, it is really interesting to see how three different spaces suggest the same prioritized compound as similar, chrysotile asbestos (CID 25 477), based on different aspects of their reactivity. It is known that these two minerals share a similar toxicity profile, due to their reactivity and capability to interact with inflammation cascade (e.g. NLRP3 inflammasome activation) (51). The second most similar compound proposed for this case study is aluminium oxide (CID 9 989 226). Even in this case, the similarity suggested is interesting because from the literature it is known that aluminium oxide shares with asbestos the same toxicity mechanism mediated by macrophage cells (52).

Results for organic compounds enlighten other interesting aspects of molecular similarity. The 2-(2-hydroxypropoxy)propan-1-ol (CID 32 881) was found to be similar to 1,1ʹ-oxydi-2-propanol (CID 8087) and 1-ethoxy-2-propanol (CID 15 287). These compounds, classified in the ‘CAMEO Chemical Reactivity Classification’ [https://cameochemicals.noaa.gov/browse/react (7 March 2023, date last accessed)] as alcohols and polyols or ethers, act as acute and chronic toxicants to different targets (53). In this case, the fingerprints bits and quantum properties of chemical spaces correctly enlightened the main features of these toxicants such as the polyols and ether functions. Fingerprints enlightened the chemical components of similarity, while quantum properties found similarity because of the same reactivity of the molecular functional groups.

For the fourth case study, 1-methyl-4-nitronaphthalene, the chemical spaces found the same molecules as the most similar, 1-methyl-5-nitronaphthalene and 1-methyl-6-nitronaphthalene (CID: 93496 and 86258), two structural isomers. It confirms again the importance of the similarity tool offering a molecule set that is similar in structural patterns and mechanism of action. Interestingly, in this case, molecular descriptors and quantum properties did not recognize the 1-methyl-6-nitronaphthalene as the most similar to the query molecule, and it is probably due to different reactivity to oxidation and subsequent toxic mechanism led by the methyl position (54).

## Conclusion

APDB is an essential resource that improves our knowledge of the existing pollutant molecules, providing an in-depth insight into their physicochemical, structural and quantum properties. The collection of pollutant molecular structures together with all the chemical characteristics provided in APDB offers scientists a unique resource for a wide view of the biochemical aspects of pollutant toxicity mechanisms. As a matter of fact, APDB is the first publicly available online database offering a complete chemical and biological annotation of air pollutants.

Moreover, derived similarities can be explored to identify similar molecules, then infer similar mechanisms of action and study potential synergistic effects in disease pathways (55). As previously discussed in the case studies section, the use of four distinctive chemical spaces for the similarity assessment between molecules allows scientists to have a view of similar compounds and therefore of putative targets/mechanism of action of the studied pollutant. The molecular properties used (fingerprints, molecular descriptors and quantum properties), in their complementarity, are capable of collecting different aspects of chemical similarity (functional groups, reactivity and connectivity) so that users can infer biochemical aspects of the toxicity behind every chemical structure. Lastly, the targets associated with the molecules can be easily retrieved and used in other fields of biomedical research, e.g. in molecular simulations, chemoinformatics approaches or toxicological and epidemiological studies (56–58).

## Supplementary Material

baad046_Supp

## Data Availability

All data and resources of APDB are freely available at https://github.com/InfOmics/APDB.
