# Supplementary material for: APDB: a database on air pollutant characterization and similarity prediction
Source: Database (Oxford). 2023 Jul 14;2023:baad046. doi: 10.1093/database/baad046 (PMC10348400; doi:10.1093/database/baad046)
Supplement: baad046_Supp [file baad046_supp.zip › suppl_data/Supplementary_Table_S1.docx]

| **Tautomer probability**: the probability of tautomeric forms |
| --- |
| **Ionization penalty**:  ionization state’s total energy penalty |
| **Ionization penalty charging**: ionization state’s energy penalty due to charged sites |
| **Ionization penalty neutral**: ionization state’s energy penalty due to neutral sites |
| **State penalty**:  state’s total energy penalty |
| **Charging adjusted penalty** |
| **Tot Q**: total formal charge |
| **Tot abs Q**: total abs formal charge |
| **Energy**: lowest conformation energy |
| **Chiralities consistent**: chiral flag |
| **Chemistry notes** |

**Table S1.** Output properties computed with LigPrep.
