# Supplementary material for: APDB: a database on air pollutant characterization and similarity prediction
Source: Database (Oxford). 2023 Jul 14;2023:baad046. doi: 10.1093/database/baad046 (PMC10348400; doi:10.1093/database/baad046)
Supplement: baad046_Supp [file baad046_supp.zip › suppl_data/Supplementary_Table_S2.docx]

| **Vibrational frequencies**: calculates frequencies, infrared (ir) intensities and thermochemical properties, such as heat capacity, entropy, enthalpy and Gibbs free energy in gas phase or in solution |
| --- |
| **Surfaces (MO, density, potential)**: provides 3D spatial information |
| **Electrostatic potential charges (ESP)**: provides the molecular electrostatic potential |
| **Mulliken Population Analysis**: provides a representation of the molecule as a set of nuclear-centered point chargers |
| **Natural Bond Orbital (NBO) Analysis**: includes analysis of the molecular dipole in terms of both NBOs and NLMOs (natural localized molecular orbitals) and natural population analysis (NPA) |
| **Multipole Moments**: computes moments with respect to the center of mass of the molecule |
| **Polarizability/hyperpolarizability**: calculates polarizabilities and first and second hyperpolarizabilities |
| **Atomic Fukui indices**: are derived from Mulliken populations for the HOMO  (highest-occupied molecular orbital) and LUMO (lowest-unoccupied molecular orbital) orbitals and are computed for both the electron density and the spin density. Jaguar provides four indices (f _NN, f_NS, F_SN, F_SS) that describe the variations in density N or spin multiplicity S relative to small variations in density N or in spin multiplicity S. A high positive value of f_NN for the HOMO indicates that the molecule can donate electrons behaving as a nucleophile. A high positive value of f_NN for the LUMO indicates that the molecule can receive electrons behaving as an electrophile |
| **Stockholder Charges**: scaled atomic densities are integrated for each atom to produce an atomic charge |

**Table S2.** Optimization and single-point energy properties [https://www.cines.fr/wp-content/uploads/2014/01/j76_user_manual.pdf, accessed 7 March 2023].
