# Supplementary material for: APDB: a database on air pollutant characterization and similarity prediction
Source: Database (Oxford). 2023 Jul 14;2023:baad046. doi: 10.1093/database/baad046 (PMC10348400; doi:10.1093/database/baad046)
Supplement: baad046_Supp [file baad046_supp.zip › suppl_data/Supplementary_Table_S3.docx]

|  | **References** |
| --- | --- |
| **Number of canonical orbitals**: number of orthogonal linear combinations of molecular orbitals | <https://www.schrodinger.com/kb/1642> |
| **HOMO and LUMO energy levels**: orbital energies of highest occupied molecular orbital (HOMO) and lowest unoccupied molecular orbital (LUMO) that describe molecular reactivity. If nucleophile, a chemical species donates an electron pair, if electrophile, it accepts an electron pair |  |
| **alpha_HOMO, alpha_LUMO, beta_HOMO, beta_LUMO**: energies of the alpha electrons and of the beta electrons in both HOMO and LUMO molecular orbitals | <https://www.cup.uni-muenchen.de/ch/compchem/pop/orb_output.html> |
| **Molecular polarizability**: measure of molecule ability to respond to an electric field and acquire an electric dipole moment | <https://www.sciencedirect.com/topics/engineering/polarizability> |
| **Dipole moment and its cartesian components** (output is in units of debye): distribution or separation of charge and polarity within a molecule | <https://pubs.acs.org/doi/10.1021/acs.jpca.1c10144> |
| **Frequencies** (output is in units of cm-1): vibrational spectra of molecules give important information on the molecular structure. The computed frequencies are: lowest frequency, highest frequency, second lowest frequency, number of negative frequencies (Note: if the geometry is a minimum, there should be no negative frequencies) | <https://www.sciencedirect.com/topics/chemistry/vibrational-frequency> |
| **Thermochemical properties** computed from vibrational frequencies (at standard temperature and pressure of 298.15 K and 1.00E+00 atm; thermochemical output is in units of kcal/mol (for H, G and U) and cal/mol K (for Cv and S) and au (for total U, total H, total G). The computed termochemical properties are: Zero point energy (ZPE, lowest possible energy of a QM system), Entropy (S), Enthalpy (H), Gibbs free energy (G), Internal energy (U), Heat capacity (Cv, change in thermodynamic energy with respect to temperature), ln(Q) (logarithm of the partition function), Total internal energy (Utot), Total enthalpy (Htot), Total Gibbs free energy (Gtot), Gas phase energy, Final energy |  |

**Table S3.** Output quantum chemical properties.
